# Maternal socioeconomic and lifestyle factors and life dissatisfaction associated with a small for gestational age infant. The Survey of Neonates in Pomerania (SNiP)

**DOI:** 10.1007/s00404-022-06598-x

**Published:** 2022-05-23

**Authors:** Guillermo Pierdant, Till Ittermann, Jennis Freyer-Adam, Ulrike Siewert-Markus, Hans Jörgen Grabe, Marcus Dörr, Matthias Heckmann, Marek Zygmunt, Anja Erika Lange, Marcello Ricardo Paulista Markus

**Affiliations:** 1 Department of Gynecology and Obstetrics, University Medicine Greifswald, Ferdinand-Sauerbruch-Straße, 17475 Greifswald, Germany; 2 Department of Study of Health in Pomerania/Clinical-Epidemiological Research, Institute for Community Medicine, University Medicine Greifswald, Greifswald, Germany; 3 DZHK (German Centre for Cardiovascular Research), partner site Greifswald, Greifswald, Germany; 4 Institute for Medical Psychology, University Medicine Greifswald, Greifswald, Germany; 5 Department of Psychiatry and Psychotherapy, University Medicine Greifswald, Greifswald, Germany; 6 Department of Internal Medicine B, University Medicine Greifswald, Greifswald, Germany; 7 Department of Neonatology and Pediatric Intensive Care, University Medicine Greifswald, Greifswald, Germany; 8 DZD (German Center for Diabetes Research), Partner site Greifswald, Greifswald, Germany

**Keywords:** Fetal growth restriction, Infant, Pregnancy, Risk factors, Small for gestational age, Socioeconomic status, Smoking in pregnancy

## Abstract

**Purpose:**

The aim is to investigate the associations of the mother’s socioeconomic and lifestyle factors and life satisfaction with the delivery of a small for gestational age (SGA) infant.

**Methods:**

Data from 4598 participants of the population-based birth cohort study Survey of Neonates in Pomerania (SniP) including comprehensive information on pregnancies, mothers, and their offspring in Western Pomerania, Germany were used in this study. The associations were analyzed using linear and logistic regression models.

**Results:**

After logistic regression analysis adjusted for height of the mother, women who delivered SGA infants, had lower education (*p* < 0.01) and smoked more frequently during pregnancy (*p* < 0.01) compared with mothers of adequate for gestational age (AGA) neonates. A mother with less than 10 years of education and one who continued smoking during pregnancy had an odds ratio (OR) of 2.23 [95% confidence interval (CI) = 1.44 to 3.46] and 2.68 (95% CI = 2.06–3.49) of having an SGA infant, respectively. There was no association between the employment of the mother (*p* = 0.28), the monthly income (*p* = 0.09), the family status (*p* = 0.80), the number of friendships outside the household that the mother would not wish to relinquish (*p* = 0.47), the number of people that she could rely on in case of an emergency (*p* = 0.75), or alcohol consumption prior to (*p* = 0.14) or during the pregnancy (*p* = 0.99) with SGA. Finally, women who delivered SGA infants were more frequently dissatisfied with their employment (*p* = 0.03) and financial status (*p* < 0.01).

**Conclusions:**

Women who delivered SGA infants had more associated socioeconomic and lifestyle risk factors and were more frequently dissatisfied with their life conditions than mothers of AGA neonates.

## Introduction

A substantial worldwide decline in maternal and neonatal mortality by nearly half has been observed in the last 2 decades [1–3]. This reduction was driven by the adoption of the world health priorities of the Millennium Development Goals (MDGs) [3], which included the offer of family planning counselling and prevention of adolescent childbearing, coverage of antenatal support, assistance of skilled health personnel at delivery and access to emergency obstetric care. On the other hand, there are still medical conditions related with infant deaths. These conditions include different disorders influencing the duration of the gestation and fetal growth that continue to have deleterious effects on the evolution of the pregnancy. Several terms are used to describe infants with low birth weights for their gestational age. These include fetal or intrauterine growth restriction (FGR/IUGR) and small for gestational age (SGA). The two terms are not synonymous. FGR/IUGR refers to the fetus who does not achieve the expected in utero growth potential due to genetic or environmental factors. SGA is defined by birth weight below the 10th percentile for gestational age (GA).

Perinatal mortality is higher in SGA infants compared with neonates born appropriate for gestational age (AGA). This association is present in both term and preterm infants [4–10]. SGA is responsible for around 22% of neonatal deaths in low- and middle-income countries [11]. It is also a cause of a wide range of short- and long-term complications [10]. SGA infants have a higher risk of morbidity and stunting in childhood, and through adulthood are predisposed to develop chronic diseases like obesity, type 2 diabetes mellitus, coronary artery disease, and stroke [12, 13]. SGA children have significantly higher systolic, diastolic and mean blood pressure than AGA children [14]. Delivering an SGA infant is also associated with maternal atherosclerotic cardiovascular disease risk [15].

Many factors affect the duration of gestation and fetal growth, and thus, the birth weight. These factors depend on the characteristics of the infant, the mother, or the physical environment and play an important role in determining the future health of the neonate [16]. SGA infants consist of constitutionally normal small neonates, as well as of intrauterine growth restricted infants due to genetic or environmental factors. Known constitutional factors that influence the occurrence of SGA include maternal height, weight, ethnicity, and parity [17] Moreover, extremes of maternal age (especially younger than 16 years or older than 40 years), multiple pregnancies, obstetric complications, maternal conditions (e.g., hypertensive disorders of pregnancies), infections (e.g., malaria), and nutritional status [18] also have an effect on the development of SGA. Paternal adverse birth outcome, particularly SGA, is a modest risk factor for SGA, independent of maternal risk status [19]. In the last decade, studies have highlighted the link between exposure to preconception stressful life events [20] and substance use such as alcohol consumption and smoking [21] with SGA pregnancies. Prenatal stress seems to lead to alterations in the brain of the newborn at multiple levels, from molecular and cellular to structural [22]. On the other hand, research regarding the associations of social support and maternal life satisfaction with SGA is scarcer. A study [23] by the Anhui Medical University in China found no association between social support and the risk of delivering an SGA infant. The Generation R Study [24] is a prospective population-based mother and child cohort in the Netherlands with 6334 mothers living in the Rotterdam area. This study that analyzes associations between maternal physical and mental health-related quality of life (HRQoL) in early, mid, and late pregnancy and adverse birth outcomes, but does not confirm the hypothesis that worse physical or mental HRQoL is associated with SGA.

The objective of this study is to analyze the relationships between mothers´ socioeconomic and lifestyle factors, social support and life satisfaction and the delivery of an infant with SGA in a birth cohort in northeastern Germany.

## Materials and methods

### Study population

#### The Survey of Neonates in Pomerania (SNiP)

**Fig. 1:**
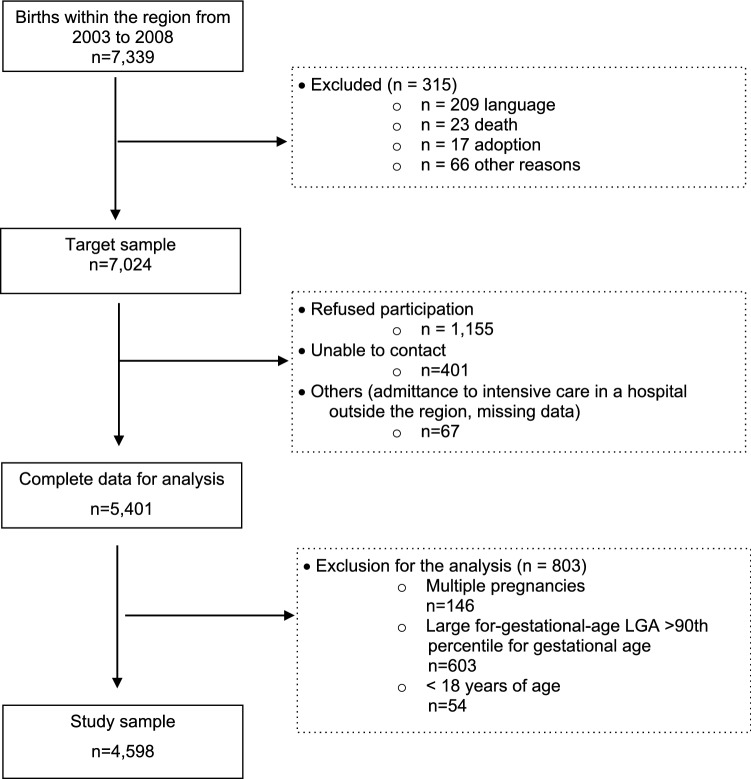
Participants’ flowchart

The present data were derived from the population-based birth cohort study Survey of Neonates in Pomerania (SNiP) [25]. The SNiP study collected comprehensive data on pregnancies, mothers, and their offspring in Western Pomerania from March 2003 until November 2008. Details have been previously reported by Ebner and colleagues, 2010 [25]. In short, all women giving birth during the study period and being registered as a resident of the study area defined by ZIP codes 17389–17999 north-eastern Germany, were asked to participate. Eligible women were asked for written informed consent. From the 7339 births within the region during the study period, 7024 mothers met eligibility criteria. Exclusion criteria comprised women with insufficient language skills, death, mothers who gave up their newborn for adoption, newborns with congenital anomalies and other reasons (Fig. 1). An SGA infant was defined as a neonate with a birth weight below the 10th percentile for gestational age based on gender-specific references for birth weight from the German perinatal survey of 2007–2011 [26]. An appropriate for gestational age (AGA) infant was defined as a neonate with a birth weight between the 10th and the 90th percentile for gestational age based on gender-specific references for birth weight from the German perinatal survey of 2007–2011 [26]. A large for gestational age (LGA) infant was defined as a neonate with a birth weight above the 90th percentile for gestational age based on gender-specific references for birth weight from the German perinatal survey of 2007–2011 [26]. Of those eligible, consent was obtained in 5401 (76.9% response). For mothers declining to participate (*n* = 1155) or women admitted to the maternity ward who we were unable to contact (*n* = 401), an anonymous minimum dataset was abstracted comprising data on the health status of the newborn, but without detailed information about environmental parameters. A non-responder analysis revealed no meaningful selection bias [25]. For the present study, we excluded mothers with multiple pregnancies (*n* = 146), younger than 18 years of age (*n* = 54), and those who delivered infants classified as large for gestational age (LGA, *n* = 603). (Fig. 1). The final analytical sample comprised 4598 mothers, aged 18 to 46 years, and their respective infants.

All mothers who participated in the study gave written informed consent. The study was approved by the ethics committee of the Medical Faculty of the Ernst-Moritz-Arndt University Greifswald and complies with the Declaration of Helsinki.

### Characteristics of the study sample

Detailed data on the health status of the mother, the newborn, the pregnancy, socioeconomic and lifestyle factors and life satisfaction were obtained via interview, standardized questionnaires and medical records. Having obtained written consent, a trained physician performed a face-to-face interview and then handed out the self-administered questionnaire. The interviewer also collected the required information from medical records. These were: (a) the prenatal care booklet (“Mutterpass”), and (b) hospital files.

The maternal age, height, prepregnancy body mass index [BMI, calculated as weight (kg)/height^2^ (m^2^)], weight gain during pregnancy in kg, number of previous pregnancies and prior pregnancy losses were obtained from the prenatal care booklet. In Germany, every pregnant woman visiting her gynecologist receives a prenatal care booklet where this information is recorded. The maternal place of birth was asked in the face-to-face interview.

### Pregnancy outcomes

The gestational age was determined from the best obstetric estimate and was recorded from the hospital files. The infant’s birth weight in g, birth length in cm, head circumference in cm, gender of the infant, mode of delivery and whether the newborn was admitted to neonatal care were collected from the hospital files.

### Maternal socioeconomic, social support and lifestyle factors variables

Women were asked about their socioeconomic status and alcohol and tobacco usage prior to and during their pregnancies through self-administered questionnaires.

The Demographic Standards for Germany [27] were used for socioeconomic variables including education, income, employment before pregnancy and family status at birth. Furthermore, questions focused on the social integration of the mothers regarding the number of people they could rely on in case of an emergency and number of friendships outside the household they would not wish to relinquish (none, one, two to three, more than three).

The Alcohol Use Disorders Identification Test-Consumption (AUDIT-C) [28, 29] was used to assess alcohol consumption. Self-reported responses were categorized into three mutually exclusive categories: (1) no maternal use before and during the pregnancy (i.e., none), (2) maternal use before the pregnancy (*former*) or (3) *current* use in the pregnancy. The Fagerström Test of Nicotine Dependence (FTND26) [30, 31] was used for smoking. Self-reported responses were categorized into four mutually exclusive categories: (1) no maternal use before and during the pregnancy (i.e., none), (2) maternal use before the pregnancy (*former*), (3) *quit during pregnancy* or (4) *current* use in the pregnancy.

### Maternal life satisfaction

Through self-administered questionnaires, the mothers reported their satisfaction with regard to their living situation in general as well as their housing, financial, leisure time, health, family and working situation and relationships with friends, neighbors and acquaintances. Satisfaction with each item was assessed with the question “To what extent are you satisfied with the item below?”, and a scale ranging from 1 to 7 (completely dissatisfied = 1, very dissatisfied = 2, dissatisfied = 3, neither dissatisfied nor satisfied = 4, satisfied = 5, very satisfied = 6, completely satisfied = 7) [32]. For a better presentation of the data, we categorized the answers into three groups (1) *dissatisfied*; which included completely dissatisfied, very dissatisfied and dissatisfied; (2) *neither dissatisfied nor satisfied*; and (3) *satisfied* which included satisfied, very satisfied and completely satisfied.

### Statistical methods

All data were stored using a Microsoft Access 2002 (Microsoft Corporation, Redmond, WA, USA) database. Descriptive data were reported as the median (25th and 75th percentiles) for continuous variables and as percentage for categorical variables stratified by birth weight for gestational age. To analyze possible differences between the subgroups (AGA and SGA) *p* values were calculated using the chi-squared test for categorical variables and the Wilcoxon rank-sum (or Mann–Whitney) tests for continuous variables.

Associations of maternal lifestyle and social factors as well as maternal life satisfaction with the delivery of an SGA infant were analyzed by logistic regression models adjusted for height of the mother, which is considered the most important constitutional factor to influence the incidence of a small for gestational age infant [33–35].

All analyses were carried out with Stata 16.1 (Stata Corporation, College Station, TX, USA). Differences between the overall totals for individual dimensions resulted from slightly different proportions of missing answers. All percentages given related to the number of questionnaires eligible for the particular dimension analyzed.

## Results

Characteristics of all study participants (*n* = 4598), stratified by birth weight for gestational age, are provided in Table 1. Mothers who delivered an SGA infant (*n* = 461) were younger, shorter, more commonly either underweight or obese and gained less weight during their pregnancy when compared to mothers who delivered an AGA infant (*n* = 4137). Women with pregnancies complicated by SGA were more frequently nulliparous. There were similar frequencies of place of birth of the mother and prior pregnancy loss between mothers of SGA and AGA infants.

**Table 1:** Maternal characteristics of the study sample stratified by birth weight for gestational age

Parameter	Total	AGA	SGA	*p* value*
*N* (%)	4598	4,137 (90.0)	461 (10.0)	
Age (years)	27 (23, 31)	27 (23, 31)	26 (22, 30)	** < 0.01**
Height (cm)	168 (164, 172)	168 (164, 172)	165 (161, 170)	** < 0.01**
Mother born in Germany (%)	97.7	97.6	98.9	0.07
Prepregnancy body mass index in kg/m^2^ (%)	22.3 (20.2, 25.1)	22.3 (20.3, 25.1)	21.7 (19.7, 24.8)	**0.01**
BMI < 18.5	7.34	6.95	10.9	
18.5 ≤ BMI < 25	66.9	67.1	65.1	
25 ≤ BMI < 30	16.9	17.1	14.9	
BMI ≥ 30	8.86	8.83	9.16	
Weight gain during pregnancy (kg)	15 (11, 19)	15 (11, 19)	14 (10, 17)	**0.02**
Parity (%)				** < 0.01**
First pregnancy	47.2	46.2	56.4	
Second pregnancy	29.5	30.0	24.3	
More than 2 pregnancies	23.4	23.8	19.3	
Prior pregnancy loss (%)	14.7	15.0	12.0	0.08

Data is median (25th, 75th percentile) or percentage

Bold values indicate statistically significant results (*p* < 0.05)

*AGA* appropriate for gestational age, *SGA* small for gestational age, *BMI* body mass index

*p values are based for difference in median on the chi-squared test for categorical variables and the Wilcoxon rank-sum (or Mann–Whitney) tests for continuous variables.

### Associations of the pregnancy outcomes with birth weight for gestational age

Overall, 7.1% of the infants were born preterm (less than 37 weeks of pregnancy) with the gestational age at delivery, gender of the infant and mode of delivery similar in SGA and AGA pregnancies. The median difference of weight between SGA and AGA infants was around 680 g (2,750 g vs 3,430 g), the median difference of length was two cm (49 cm vs 51 cm) and the median difference of head circumference was one cm (34 cm vs 35 cm) between both groups. Moreover, SGA infants were twice as often admitted to neonatal care compared to infants born AGA (Table 2).

**Table 2:** Pregnancy outcomes of the study sample stratified by birth weight for gestational age

Parameter	Total	AGA	SGA	*p* value*
*N* (%)	4598	4137 (90.0)	461 (10.0)	
Gestational age in weeks at delivery (%)				0.71
< 32 weeks	1.04	1.02	1.30	
32 ≤ weeks < 37	6. 09	6.21	4.99	
37 ≤ weeks < 41	91.6	91.5	92.4	
≥ 41 weeks	1.26	1.26	1.30	
Birth weight (g)	3370 (3050, 3650)	3430 (3160, 3680)	2750 (2530, 2920)	** < 0.01**
Birth length (cm)	51 (49, 52)	51 (50, 52)	49 (47, 50)	** < 0.01**
Head circumference (cm)	35 (34, 36)	35 (34, 36)	34 (33, 34)	** < 0.01**
Mode of delivery (%)				0.42
Vaginal	71.7	72.1	68.4	
Caesarean section	24.2	23.9	26.8	
Instrumental	4.10	4.03	4.79	
Gender of the infant (%)				0.88
Male	53.3	53.3	52.9	
Female	46.7	46.7	47.1	
Admission to neonatal care (%)	7.16	6.45	13.5	** < 0.01**

Data is median (25th, 75th percentile) or percentage

Bold values indicate statistically significant results (*p* < 0.05)

*AGA* appropriate for gestational age, *SGA* small for gestational age

*p values are based for difference in median on the chi-squared test for categorical variables and the Wilcoxon rank-sum (or Mann–Whitney) tests for continuous variables.

### Associations of the maternal socioeconomic, social support and lifestyle factors with birth weight for gestational age

Women who delivered SGA infants had fewer years of education when compared with mothers of AGA neonates. While one quarter of them had fewer than 10 years of education, twice as many of the women who delivered AGA infants held university degrees. After logistic regression analysis adjusted for the height of the mother, a mother with less than 10 years of school education had an OR of 2.23 [95% confidence interval (CI) = 1.44 to 3.46] of having an SGA infant. While a high proportion of all pregnant women continued smoking during their pregnancies (around 21%), the prevalence of current smoking of the SGA pregnancies was twice as frequent when compared to AGA pregnancies. After logistic regression analysis adjusted for the height of the mother, a mother who continued to smoke during pregnancy had an OR of 2.68 (95% CI = 2.06–3.49) of having an SGA infant. On the other hand, there was no association between the employment or unemployment of the mother before pregnancy (OR = 1.17; 95% CI = 0.88–1.57), the monthly income (OR = 0.98; 95% CI = 0.95–1.00), the family status at birth (OR = 1.06; 95%CI = 0.67–1.70), the number of friendships outside the household that the mother would not wish to relinquish (OR = 1.29; 95% CI = 0.65–2.56), the number of people that the mother could rely on in case of an emergency (OR = 1.20; 95% CI = 0.64–2.25) and alcohol consumption before (OR = 0.78; 95% CI = 0.55–1.09) or during the pregnancy (OR = 1.00; 95% CI = 0.69–1.44) with the delivery of an SGA infant (Table 3).

**Table 3:** Adjusted^a^ odds ratio (95% CI) for small for gestational age according to maternal socioeconomic, social support, and lifestyle factors

Parameter	Total	AGA	SGA	OR (95% CI)	*p* value*
*N* (%)	4598	4137 (90.0)	461 (10.0)		
Educational status					
University degree	12.5	13.1	7.59	1	
> 10 years	19.3	19.8	14.7	1.22 (0.77–1.93)	0.39
10 years	52.8	52.4	56.4	1.75 (1.18–2.60)	**0.01**
< 10 years	15.3	14.7	21.3	2.23 (1.44–3.46)	** < 0.01**
Employment of the mother before pregnancy					
Full-time	42.1	42.4	39.9	1	
Part-time	16.9	17.3	13.1	0.77 (0.52–1.13)	0.18
Leave of absence	6.74	6.86	5.65	0.82 (0.47–1.42)	0.48
Training	5.55	5.27	8.13	1.53 (0.94–2.50)	0.08
Unemployed	28.7	28.2	33.2	1.17 (0.88–1.57)	0.28
Equivalent income^b^	1,060 (618, 1,565)	1,060 (618, 1,587)	795 (441, 1,502)	0.98 (0.95–1.00)	0.09
Family status at birth					
Living with a partner	95.0	95.1	94.8	1	
Alone	4.96	4.93	5.25	1.06 (0.67–1.70)	0.80
Number of friendships outside the household that the mother would not wish to relinquish					
More than three	53.1	53.5	49.1	1	
Two or three	36.9	36.7	38.0	1.10 (0.84–1.43)	0.50
One	7.07	6.81	9.41	1.44 (0.92–2.24)	0.11
None	3.00	2.95	3.48	1.29 (0.65–2.56)	0.47
Number of people that the mother could rely on in case of an emergency					
More than three	70.0	70.5	65.3	1	
Two to three	25.8	25.4	29.9	1.25 (0.95–1.64)	0.11
One	3.51	3.44	4.17	1.20 (0.64–2.25)	0.56
None	0.65	0.65	0.69	1.27 (0.29–5.66)	0.75
Alcohol use					
Never	13.9	13.7	16.0	1	
Former	56.2	56.8	51.0	0.78 (0.55–1.09)	0.14
Current use in the pregnancy	29.9	29.5	33.0	1.00 (0.69–1.44)	0.99
Cigarette use					
Never	39.2	40.3	28.8	1	
Former	11.7	12.2	7.89	0.91 (0.60–1.38)	0.67
Quit during pregnancy	28.2	28.5	25.5	1.26 (0.95–1.68)	0.11
Current	20.9	19.0	37.9	2.68 (2.06–3.49)	** < 0.01**

Data is median (25th, 75th percentile) or percentage

Bold values indicate statistically significant results (*p* < 0.05)

*AGA* appropriate for gestational age, *SGA* small for gestational age

*p value is based on the Wald test. ﻿

^a^Logistic regression adjusted for maternal height

^b^The OR were calculated for an increase of 100€ in income

### Associations of life satisfaction with birth weight for gestational age

Women who delivered SGA infants were more frequently dissatisfied with their employment (OR = 1.39; 95% CI = 1.04–1.87) and financial status (OR = 1.83; 95% CI = 1.37–2.45) and with their living situation in general (OR = 2.22; 95 CI = 1.01–4.89). On the other hand, there were no associations between the mother´s satisfaction with their housing situation (OR = 1.43; 95% CI = 0.92–2.24), leisure time (OR = 1.07; 95% CI = 0.61–1.91), health situation (OR = 1.67; 95% CI = 0.86–3.24), family situation (OR = 1.77; 95% CI = 0.93–3.36) and relationships with friends, neighbors and acquaintances (OR = 1.32; 95% CI = 0.62–2.82) and the birth weight for gestational age (Table 4).

**Table 4:** Adjusted^a^ odds ratio (95% CI) for small for gestational age according to maternal life satisfaction

Parameter	Total	AGA	SGA	OR (95% CI)	*p* value*
*N* (%)	4598	4137 (90.0)	461 (10.0)		
Satisfaction with the housing situation					
Satisfied	86.0	86.7	79.7	1	
Neither satisfied nor dissatisfied	7.32	6.90	11.6	1.71 (1.14–2.56)	**0.01**
Dissatisfied	6.66	6.42	8.77	1.43 (0.92–2.24)	0.12
Satisfaction with the financial situation					
Satisfied	50.7	52.0	38.6	1	
Neither satisfied nor dissatisfied	24.1	23.7	27.4	1.48 (1.09–2.01)	**0.01**
Dissatisfied	25.3	24.3	34.0	1.83 (1.37–2.45)	** < 0.01**
Satisfaction with the leisure time					
Satisfied	82.1	82.3	80.9	1	
Neither satisfied nor dissatisfied	13.0	12.8	14.2	1.13 (0.79–1.62)	0.49
Dissatisfied	4.93	4.93	4.96	1.07 (0.61–1.91)	0.81
Satisfaction with the health situation					
Satisfied	92.2	92.3	91.3	1	
Neither satisfied nor dissatisfied	5.33	5.38	4.90	0.90 (0.51–1.59)	0.71
Dissatisfied	2.44	2.28	3.85	1.67 (0.86–3.24)	0.13
Satisfaction with the family situation					
Satisfied	94.3	94.4	93.0	1	
Neither satisfied nor dissatisfied	3.29	3.34	2.81	0.81 (0.39–1.70)	0.58
Dissatisfied	2.45	2.25	4.21	1.77 (0.93–3.36)	0.08
Satisfaction with the working situation					
Satisfied	62.5	63.4	54.7	1	
Neither satisfied nor dissatisfied	14.2	13.9	16.8	1.33 (0.93–1.90)	0.11
Dissatisfied	23.3	22.7	28.5	1.39 (1.04–1.87)	**0.03**
Satisfaction with the relation to friends, neighbors and acquaintances					
Satisfied	94.3	94.4	93.3	1	
Neither satisfied nor dissatisfied	3.54	3.50	3.89	1.05 (0.55–1.99)	0.89
Dissatisfied	2.21	2.14	2.83	1.32 (0.62–2.82)	0.47
Satisfaction with the living situation in general					
Satisfied	93.0	93.2	91.2	1	
Neither satisfied nor dissatisfied	5.49	5.44	5.96	1.11 (0.66–1.88)	0.68
Dissatisfied	1.47	1.32	2.81	2.22 (1.01–4.89)	0.05

Data is percentage

Bold values indicate statistically significant results (*p* < 0.05)

*AGA* appropriate for gestational age, *SGA* small for gestational age

*p value is based on the Wald test.

^a^Logistic regression adjusted for maternal height

## Discussion

In this population-based cohort, we found numerous associations between maternal socioeconomic and lifestyle factors and life satisfaction and the risk of delivering an SGA infant. Women who delivered SGA infants were younger, shorter, more commonly both underweight and obese, gained less weight during their pregnancy and were more frequently nulliparous. Beyond these already known constitutional risk factors [34], we found that in women who had SGA infants, the number of years of education was lower and the proportion of active smokers during pregnancy was twice as high. Contrary to the association between SGA and school education, we found that the monthly income did not show an effect on the chance of having a SGA infant. We believe the explanation for these results is that the monthly income is a potentially endogenous variable. It might correlate with unobservable characteristics and the parental education effect is transmitted to a certain degree through income. We believe these are important aspects that require further studies [36].

Moreover, the mothers of SGA infants were more frequently dissatisfied with their work, financial and living situation in general. We believe that these associations might explain the previously described social determinants of health during a pregnancy wherein social factors can influence the fetal development and are associated with the birth of an SGA infant.

### In the context of the published literature

Prior studies [20, 21, 23, 34, 37, 38] have demonstrated an association of specific maternal demographic and behavioral factors with the delivery of an SGA infant. However, as far as we know, this is the first population-based study that analyzed the associations of satisfaction with the living situation in general as well as individual dimensions such as housing, financial, leisure time, health, family and working situation and relations with friends, neighbors and acquaintances, with an incident SGA birth.

Short stature, [34] low weight before pregnancy, [34, 38, 39] low weight gain during pregnancy, [34, 38] lower educational status, [23] lower income [23] and maternal smoking [34, 38] are factors that have previously been consistently associated with the risk of delivering an SGA infant and are in line with our results. Other risk factors like maternal age, [34, 38, 40] socioeconomic factors [34, 38, 40] and alcohol consumption [34, 38, 40] have been inconsistently identified across studies.

Social support was assessed by the Chinese Revised Edition of the Social Support Scale in a study [23] with 1800 women aged 20–34 years who delivered after 32 weeks’ gestation in the Anhui Medical University in China. There was no association between social support and the risk of delivering an SGA infant. These results are consistent with our results.

Furthermore, an analysis from the consecutive pregnancies study, [34] a retrospective cohort study which included 27,077 women who were nulliparous and had singleton deliveries, also showed that race/ethnicity, marital or insurance status or alcohol consumption were not associated with SGA.

Contrary to our study, where we did not find an association between alcohol consumption and SGA birth, a case–control study [41] with 518 pairs of pregnant Spanish women found that alcohol consumption of less than < 4 g/day appeared to exert a protective effect against the delivery of an SGA newborn in comparison to women reporting no alcohol intake during the pregnancy (OR = 0.62; 95% CI = 0.43–0.88). A possible explanation for the different results might be related to biased selection or residual confounding.

In contrast to the results of our study, the Generation R Study [24] did not confirm the hypothesis that worse physical or mental health-related quality of life (HRQoL) is associated with adverse birth outcomes including SGA. HRQoL is a measure of the personal perception of the value and quality of life as influenced by disease, injury, and treatment. The HRQoL was measured using the 12-item Short Form Survey (SF-12) that covers eight areas: physical functioning, role limitations due to physical problems, bodily pain, general health, vitality, social functioning, role limitations due to emotional problems, and perceived mental health. Physical and mental component summary scores were calculated. A possible explanation for the results might be related to the relatively healthy and educated (one quarter of the women had a university degree) women enrolled in the Generation R Study when compared to the general Dutch population.

A study examined child hair cortisol concentration (HCC) and maternal stress during pregnancy in a low-income sample consisting of 77 healthy mother-children pairs [42]. Maternal stress was measured with the perceived stress scale during pregnancy. Child HCC was measured approximately four years later. Regression analysis revealed that child HCC was not significantly predicted by maternal perceived stress.

A study in one hospital in Taiwan [43] with 198 pregnant women without complications assessed the maternal quality of life monthly, beginning between 25 and 29 gestational weeks, until 1 month postpartum. The Duke Health Profile (DUKE) was used to measure quality of life. The DUKE is a 17-item, three-point measure of the physical, mental, social, general, and perceived health. The results of the study are consistent with our results. They suggest that poor maternal physical and social health at late pregnancy could predict preterm birth and that low maternal health scores at earlier pregnancy tended to predict infants born with a low birthweight.

Many factors affect the fetal growth and thus the birth weight for gestational age. They relate to the infant, the mother, and the physical environment and play an important role in determining the future health of the infant [16]. We believe that the social and psychological determinants that are associated with a healthy pregnancy, which we are able to document as an adequate birth weight for the gestational age, vary depending on the population analyzed [44]. This might help to explain why the incidence and prevalence of SGA varies between different analyzed populations [11]. For example, the incidence of SGA in developing countries is twice as high compared to developed countries (20 percent vs. 10 percent) [11, 45]. Our results suggest the importance of documenting and analyzing not only the already known constitutional risk factors, but also the possible maternal life satisfaction aspects in relation to the incidence of SGA births. SNiP comprises the population of a sparsely populated area in northeastern Germany, a population still adapting to social and economic hardships since the German reunification in 1990 [25]. The analyzed population of Western Pomerania lives in a region with higher unemployment and poverty risk rates than the national average [46, 47] and presents the lowest life expectancy in Germany [46, 47]. Although in the past century, neonatal mortality in Germany decreased similarly to other Western nations, these changes were less pronounced in East Germany [48].

If a correlation between psychosocial risk factors in pregnant women and birth weight is confirmed, it might be possible to develop screening tools to identify women with increased risk for developing a pregnancy with SGA fetuses and provide the necessary support to avoid this complication.

### Strengths and limitations

The main strength of this study is the use of a large sample which includes the majority of the births in a specific population over a period of time. Furthermore, we used specific birth weight charts for the analyzed population. This reduces the risk of potential misclassification as birth weight charts influence the classification and incidence of SGA [49].

Study limitations include the difficulty in obtaining valid and reliable data on alcohol intake and smoking during pregnancy. Although this was mitigated by the use of validated questionnaires, the AUDIT-C [28] and the FTND26, [30] misclassification must always be considered, especially during pregnancy, because of socially desirable answers. Moreover, the questionnaires in our study were completed between the delivery and hospital discharge so that memory bias cannot be ruled out. Another limitation of the study is the exclusion of participants with missing data, which might limit the generalizability of the results to some extent. There are also other potential confounding variables that were not included in the analyses, for example, diet related factors. The associations could be cofounded by other unfavorable lifestyle or medical factors.

## Conclusions

While previous studies have shown the relative influence of environmental and genetic factors that explain the variation in birth weight, our findings showed an association of maternal lower educational status, smoking and life dissatisfaction with delivery of an SGA infant. Future studies should analyze whether interventions designed to support women exposed to these risk factors might avoid the occurrence of SGA births and their complications.

## Data Availability

The data of the SNIP-study is publicly available via https://www.fvcm.med.uni-greifswald.de/. This is a data repository where any researcher can register and find data dictionary as well as an online application tool for getting access to data. Upon an application by registered users, the Research Cooperation Community Medicine (RCC) of the University of Greifswald, Germany, which is funded by the Federal Ministry of Education and Research (grant no. ZZ 96030) decides on granting access to the data based on scientific guidelines.
